# Study on the Properties of Foamed Mixture Lightweight Soil Prepared from Waste Dredged Soil for Ecological Floating Landscapes

**DOI:** 10.3390/ma19122512

**Published:** 2026-06-10

**Authors:** Xujiang Xia, Xiang Chen, Ning Zhuang, Wenrui Xiao, Yalin Wang

**Affiliations:** 1 College of Harbor, Coastal and Offshore Engineering, Hohai University, Nanjing 210098, China; 18260635129@163.com (X.X.); 13203232189@163.com (W.X.); wangyalin1212@hhu.edu.cn (Y.W.); 2 Key Laboratory of Ministry of Education for Coastal Disaster and Protection, Hohai University, Nanjing 210098, China; 3 CCCC Shanghai Dredging Co., Ltd., Shanghai 200136, China; 15921524735@139.com

**Keywords:** foamed mixture lightweight soil, optimal mixing ratio, waste dredged soil, expansion agent, fiber

## Abstract

This paper develops foamed mixture lightweight soil (FMLS) using dredged soil for ecological floating landscapes applications, focusing on key performance indices including dry density, compressive strength, splitting tensile strength, water absorption, and fluidity. Orthogonal experiments determined the optimal mix ratio, while CaO expansion agent, MgO expansion agent, polypropylene fiber (PPF), and basalt fiber (BF) were employed to modify material properties. The microstructural mechanisms of FMLS before and after modification were characterized by scanning electron microscopy (SEM). The results show that FMLS achieves optimal comprehensive performance at a cement-to-sand ratio of 0.4, foam content of 10%, and water-to-sand ratio of 0.35, with all parameters conforming to technical specifications. The optimal dosage for both CaO and MgO expansion agents is 5%, PPF is 0.3% and BF is 0.5%, respectively. MgO expansion agent and PPF demonstrate superior suitability for floating landscapes due to enhanced pore-filling efficiency and crack-bridging effects by SEM. Finally, correlation analysis further indicates that the water–binder ratio critically governs the strength characteristics of FMLS. This paper not only provides a new direction to promote the effective use of dredged soil resources, but also provides new ideas for carrier materials for ecological floating landscapes.

## 1. Introduction

In recent years, the advancement of ecological landscape engineering has led to the widespread adoption of ecological floating landscapes, offering benefits such as water purification, landscape enhancement, and habitat creation [1]. The ideal substrate for such landscapes requires characteristics like low density, high tensile strength, and durability [2]. However, conventional materials not only deplete resources but also struggle to achieve a balance between lightweight properties and mechanical strength. Moreover, inland waterway dredging projects, such as those in the Yangtze River, generate substantial amounts of dredged soil [3,4,5]. Conventional methods for treating dredged soil, given its intricate physical and chemical properties and associated pollution risks, have led to notable ecological and economic challenges [6,7,8,9]. Therefore, promoting the efficient resource utilization of dredged soil has become an important way to alleviate environmental pressure and promote the circular economy.

Dredged soil exhibits susceptibility to cracking and inadequate strength due to its loose pore structure and inherently low strength when used in construction materials [10]. Consequently, its application is severely restricted, typically limited to non-structural or ancillary components such as subgrade cushioning [11]. Ecological floating landscapes impose comparatively low strength demands on materials, presenting a promising avenue for the extensive utilization of dredged-soil resources. Dredged soil as a raw material for producing foamed mixture lightweight soil offers a novel approach to addressing challenges related to resource depletion, environmental impact, and engineering specifications.

Foamed mixture lightweight soil (FMLS) is an ideal material for non-structural applications such as subgrade cushioning, characterized by low density, high compressive strength, and excellent fluidity [12,13,14]. As early as the 1980s, Japan conducted many laboratory and field tests on FMLS and recommended FMLS as a practical material in the field of civil engineering [15]. FMLS incorporates air bubbles into a blended soil slurry [16], and these entrapped bubbles create numerous micropores, reducing the material density after solidification. The pre-foaming method, compared with mechanical foaming, has become predominant globally due to its superior control over density, enhanced fluidity, and ease of construction. This method allows for the tailored preparation of FMLS with varying proportions and densities to meet specific engineering requirements, such as embankment widening engineering [17]. Inappropriate material selection or mixing ratios can lead to significant shrinkage, compromising construction quality because FMLS is a highly porous medium designed to be placed without compaction. In the past engineering cases, FMLS primarily utilized natural sand and cement, with fly ash, slag, and silica fume as additives to enhance strength, durability, and impermeability [16,18]. Zhang et al. (2015) found that the incorporation of a certain dosage of slag in fly ash increased the compressive strength of FMLS [19]. She et al. (2018) showed that fly ash can significantly increase the compressive strength of FMlLS but affects the water absorption and drying shrinkage [20]. Mashifana et al. (2021) found that slag had a facilitating effect by balancing the ionic charge [21]. Therefore, the addition of moderate amounts of silica fume, fly ash, and slag can improve the properties of FMLS. However, there are studies indicating that the high water absorption of fly ash, slag, and silica fume can detrimentally impact the stability of ecological floating landscapes, so these modifiers are unsuitable for such applications [22,23].

Therefore, optimized mix ratios and alternative modification strategies are significant for preparing FMLS suitable for ecological floating landscapes. Orthogonal testing is a prevalent and efficient method for optimizing mix proportions, minimizing the number of tests required while effectively evaluating the combined effects of key factors [24,25] such as water–sand ratio, binder content, and foam content [26]. Incorporating expansive agents and fibers represents a crucial method for enhancing concrete properties, in addition to adding fly ash and lime [27,28]. Expansive agents form crystals via hydration reactions, filling soil pores and generating micro-expansion stress to counteract drying shrinkage [29]. Fibers establish a three-dimensional network that hinders the initiation and propagation of microcracks, significantly enhancing tensile toughness, crack resistance, and ductility [30]. The above modification mechanism theoretically mitigates the adverse effects associated with the high-water absorption of fly ash and lime, offering a more suitable approach for enhancing FMLS performance in ecological floating landscapes. However, the actual efficacy of this method requires experimental validation.

To provide a clearer comparison between existing solutions and the approach proposed in this study, representative studies on lightweight geomaterials, dredged-soil reutilization, fiber-reinforced lightweight soils, expansive-agent-modified cementitious materials, and ecological floating landscape substrates are systematically summarized. The corresponding research gaps are presented in Table 1.

Based on the research gaps summarized in Table 1, the present study developed a modified FMLS using waste dredged soil as the main solid raw material for ecological floating landscape applications. Unlike conventional FMLS studies that mainly focus on backfilling or subgrade engineering, this work addresses the lack of a systematic modification strategy for dredged-soil-based FMLS specifically designed to meet the coupled requirements of ecological floating landscapes, including low density, adequate compressive and splitting tensile strength, reduced water absorption, and long-term stability in aquatic environments. To fill this gap, an orthogonal experimental design was first used to determine the optimal basic mixture ratio. Then, CaO and MgO expansive agents, polypropylene fiber, and basalt fiber were introduced to evaluate their effects on density, strength, water absorption, fluidity, and microstructure. The novelty of this work lies in proposing a modified FMLS system that combines dredged-soil reutilization with pore-structure regulation and fiber/expansive-agent reinforcement, thereby providing a lightweight and sustainable carrier material for ecological floating landscape construction.

## 2. Materials and Methods

### 2.1. Experimental Materials

The test raw material is dredged soil, collected from the Hengsha Shoal in the lower reaches of the Yangtze River in Shanghai. The dredged soil’s PH value is 7.25, and the major composition is shown in Table 2. Dredged soil contains 63.73% SiO_2_, 14.33% Al_2_O_3_, and 1.36% Na_2_O. SiO_2_ and Al_2_O_3_ help to form a strong skeletal structure and enhance the overall stability of the soil. Na_2_O not only helps to regulate the chemical properties of the soil and enhances the stability of the foam, but also may provide hydroxide radicals for the hydration reaction. OPC (Ordinary Portland cement) with a strength grade of 42.5 MPa, produced by Anhui Conch Cement Co., Ltd. (Wuhu, China).

A comparative analysis was conducted on the efficacy of different kinds of foaming agents commercially available, as detailed in Table 3. Plant protein foaming agent was selected from its superior performance in foaming, foam stability, durability, and environmental sustainability to ensure the desired properties of the FMLS.

The expansion agents used in the test are the CaO expansion agent produced by Tianjin ZhiYuan Reagent Co., Ltd. (Tianjin, China), and the MgO expansion agent produced by Fuchen Reagent Co., Ltd. (Tianjin, China). The purpose of joining the expansion agent is to decrease the drying shrinkage of the FMLS [43] and provide a positive effect on the strength of the FMLS [44]. The basic information of the expansion agent is shown in Table 4.

The kind of fiber used in the test is polypropylene fiber (PPF) and basalt fiber (BF). PPF exhibits low relative density and excellent corrosion resistance [45,46,47]. During mixing, the PPF’s smooth surface facilitates dispersion, leading to the formation of a fiber network structure that enhances soil uniformity. The smooth surface of PPF facilitates the formation of a fiber network structure during mixing and improves FMLS homogeneity. BF is recognized for its high tensile strength, elastic modulus, and aging resistance, as well as its excellent compatibility with cement-based materials [48,49]. Table 5 presents the performance characteristics of fiber materials.

### 2.2. Specimens’ Preparation and Procedure

**Figure 1:**
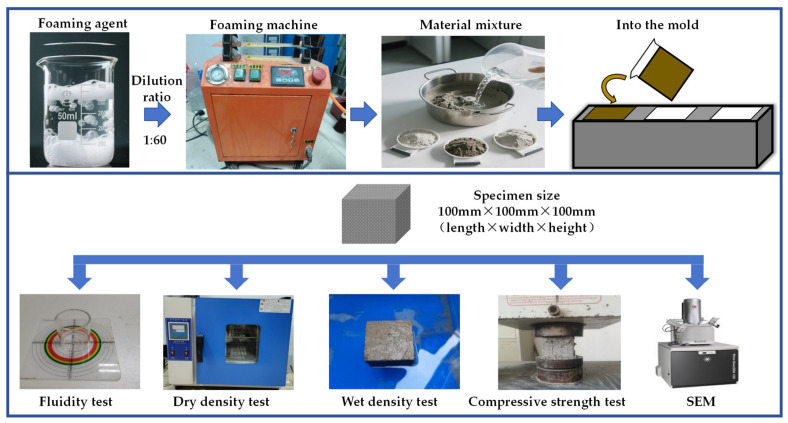
The flow chart of preparation and detection of the FMLS.

Figure 1 shows the flowchart for preparing and testing FMLS. Preparation involved four main steps: (1) An air compressor diluted the foaming liquid 60 times to generate sufficient foam, which was then stopped. (2) Dredged soil, Portland cement, and water were weighed according to the predetermined ratio. Water was added first to the mixing vessel, followed by Portland cement and dredged soil. The mixture was stirred to homogeneity. The pre-generated foam was then incorporated into the cement-dredged soil slurry and mixed thoroughly. (3) After achieving uniform mixing, fluidity was tested according to Chinese standard CJJ/T 177-2012 [50]. (4) The specimens were demolded, sealed in plastic bags, and transferred to a standard curing chamber for the specified duration after 24 h.

The procedure entails incorporating foam into a cement-based gelling slurry to form the lightweight soil mixture, followed by casting, molding, and curing. A series of tests is carried out to test the specimens after curing.

The test program was organized into three stages: basic mixture optimization, expansive-agent modification, and fiber modification. The basic mixture optimization included pre-test design groups (A-1–A-5). There were also nine orthogonal groups (B-1–B-9). The expansive-agent modification included six groups (C-1–C-6), and the fiber modification included fourteen groups (D-1–D-14). For each mixture group, all quantitative measurements were conducted in triplicate unless otherwise stated. Specifically, fluidity was measured three times for each fresh mixture; dry density, wet density, and water absorption were determined using three parallel specimens; and compressive strength and splitting tensile strength were tested using three independent specimens at each curing age of 7 d, 14 d, and 28 d. The average value of the three repeated measurements was used as the representative result. SEM observation was performed on representative specimens to support the interpretation of the macroscopic results.

### 2.3. Test Methods

The fluidity test was conducted according to the Chinese standard CJJ/T 177-2012. Prior to testing, the inner surfaces of the hollow plastic cylinder (80 mm inner diameter, 80 mm length) and the plastic plates (400 mm × 400 mm) were thoroughly cleaned. A damp cotton cloth was used to remove surface impurities and residues, ensuring optimal test conditions and compliance with the standard. The FMLS was slowly poured into the cylinder. When filled to approximately two-thirds of its height, the mixture was gently stirred using a stirring bar to ensure uniform distribution. After filling and scraping the surface level, the cylinder was vertically lifted and the mixture allowed to stand for 1 min. The diameter of the resulting soil patty was accurately measured using an electronic vernier caliper. This measurement was defined as the fluidity of the FMLS. To ensure reliability, the test was repeated three times, and the arithmetic mean of the measurements was recorded as the representative fluidity value.

The dry density was determined in accordance with the Chinese standard GB/T 11969-2020 [51]. Three specimens from each group were subjected to a drying process in an electrothermal blast drying oven after a 28-day curing period. The specimens underwent a staged thermal treatment: (1) They were incubated at a temperature of (60 ± 5) °C for 24 h; (2) They were followed by an additional 24 h incubation at (80 ± 5) °C; (3) They were baked at (105 ± 5) °C until reaching a constant weight (*M*_0_). Constant weight was defined as the difference in mass measured twice by an electronic balance, not exceeding 5% of the mass of the specimen at an interval of 4 h during drying. The average of the three specimens’ measurement results was taken as the representative data for the dry density. The dry density formula is shown in Equation (1).

(1) $$\rho =\frac{M_{0}}{V}\times 10^{3}$$

where *ρ* denotes the dry density of the specimen (g/cm^3^), *M*_0_ is the mass of the specimen (g), and *V* refers to the volume of the specimen (cm^3^).

The wet density was determined according to the Chinese standard GB/T 11969-2020. Three oven-dried specimens (constant mass achieved) were selected from each group and immersed in water maintained at (20 ± 5) °C. Water was added to initially submerge the specimens to one-third of their height. After 24 h, the water level was raised to two-thirds of the specimen height. After a further 24 h, sufficient water was added to cover the specimens by more than 30 mm. Following an additional 24 h immersion period, the specimens were removed from the tank. Surface moisture was blotted off with a damp cloth, and the specimens were immediately weighed on a precision electronic scale. The measured mass of each specimen was recorded as (*M_g_*). The average of these values was taken as the representative wet density. The formulae for water absorption rate and wet density are given in Equations (2) and (3).

(2) $$W_{R}=\frac{M_{0}-M_{g}}{M_{0}}\times 100\%$$

(3) $$\rho_{0}=\frac{M_{g}}{V}$$

where *W_R_* denotes the water absorption rate (%), *M_g_* is the mass of the specimen after water absorption (g).

Both compressive strength and splitting tensile strength were determined in accordance with the Chinese standard GB/T 11969-2020 using an SHT4305 microcomputer-controlled electro-hydraulic servo universal testing machine. Before testing, the loading system was checked to ensure stable and continuous loading. The tests were conducted under load-control mode, with loading rates of 0.5 kN/s for compressive strength and 0.25 kN/s for splitting tensile strength until specimen failure. The peak failure load was recorded automatically by the testing system. For each mixture group and curing age, three parallel specimens were tested, and the average value was taken as the representative strength. The compressive strength and splitting tensile strength formulae are given in Equations (4) and (5).

(4) $$f_{cc}=\frac{P}{A}$$

(5) $$f_{ts}=\frac{2P_{ts}}{\pi A_{ts}}$$

where *f_cc_* is the compressive strength (MPa), *P* denotes the load value at the moment of damage (N), *A* refers to the compressive area of the specimen (mm^2^), *f_ts_* is the splitting tensile strength (KPa), *P_ts_* denotes the load value at the moment of damage (N), and *A_ts_* refers to the specimen split surface area (mm^2^).

In this study, all quantitative results are presented as the arithmetic mean of three repeated measurements or three parallel specimens. The repeated-test design was used to reduce random experimental error and to ensure the reliability of comparisons among different mixture groups. Scanning electron microscopy (SEM) was performed to observe the microstructural morphology of FMLS specimens. The SEM observation procedure was carried out with reference to the general principles of microstructural characterization of cement-based materials reported in previous studies and the operating requirements of the Hitachi SU8010 scanning electron microscope. After curing, representative fragments were collected from the interior of selected specimens, dried to remove free water, mounted on aluminum stubs using conductive adhesive, and sputter-coated with gold to improve electrical conductivity. The prepared samples were then observed under high-vacuum conditions using a Hitachi SU8010 SEM at different magnifications.

### 2.4. Mixing Ratio Design

**Figure 2:**
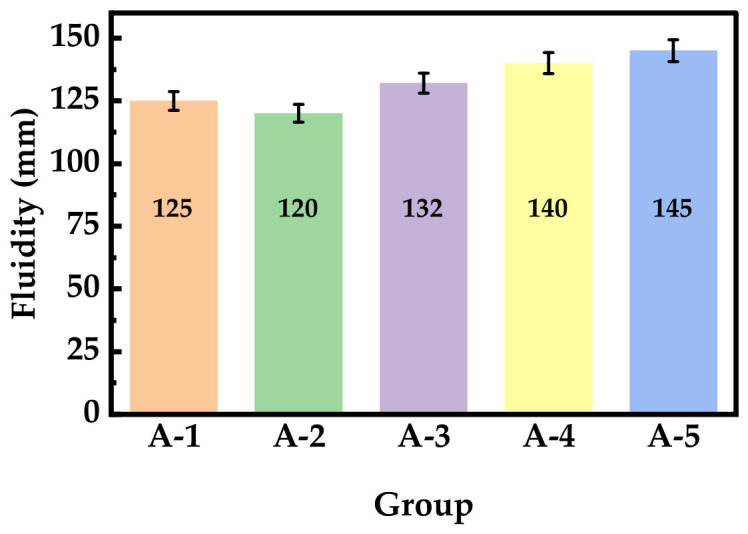
Comparative diagram of pre-test fluidity.

The cement–sand ratio and water–sand ratio ranges were established by pre-test, as detailed in Table 6. Slurry fluidity was determined in accordance with the Chinese standard GB/T 2419-2005 [52]. Figure 2 illustrates the fluidity results for each test group.

As Figure 2 illustrates, slurry fluidity peaked at water–sand and cement–sand ratios of 0.4, while decreasing these ratios to 0.25 and 0.3, respectively, causes significant fluidity deterioration. To optimize FMLS proportions, we conducted orthogonal experiments focusing on three key performance factors: cement–sand ratio (factor A), foam volume content (factor B), and water–sand ratio (factor C). Factor levels were established based on engineering experience and preliminary tests, as shown in Table 7. The foam content was added according to a percentage of the total volume. The different mix proportion schemes are presented in Table 8 (A1 denotes the first level of factor A; that is, the value of A1 is 0.3), where the foam content is expressed as a percentage of the total volume. Optimal proportions were identified by analyzing density, water absorption, and strength properties across formulations.

Following the determination of the optimum mixture ratio for the FMLS, the effects of various expansion agents and fiber contents on its properties were investigated through the incorporation of CaO expansion agent, MgO expansion agent, PPF, and BF.

## 3. Results and Discussion

### 3.1. Analysis of the Optimum Mixture Ratio Test Results

#### 3.1.1. Dry–Wet Density and Water Absorption Rate

**Figure 3:**
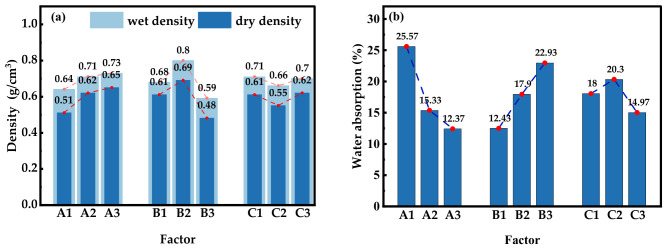
Calculation of extremum difference analysis: (**a**) density; (**b**) water absorption.

The results from the orthogonal tests presented in Table 9 were analyzed using the extremum difference analysis method. In this method, *K_jm_* denotes the mean of the experimental indices corresponding to the *m*-th level of the *j*-th factor, and *R_j_* is defined as the range among these *K_jm_* values for factor *j* [53]. Due to the balanced nature of the orthogonal design, the *K* values for different levels of a factor are directly comparable. Thus, the magnitude of the effect of a factor on the experimental results and the range of its effects can be determined effectively by directly comparing the K values corresponding to each level of that factor. The specific analysis results are summarized in Figure 3.

Figure 3a depicts the temporal evolution of dry and wet densities in FMLS across different factor levels. From level A1 to A3, wet density increases from 0.64 g/cm^3^ to 0.73 g/cm^3^, while dry density rises from 0.51 g/cm^3^ to 0.65 g/cm^3^, indicating a positive correlation between factor A and density enhancement. Notably, level B2 yields peak densities of 0.80 g/cm^3^ (wet) and 0.69 g/cm^3^ (dry), highlighting its significant impact on density augmentation. For factor C, the C1 level achieves the highest dry density, outperforming other levels. Figure 3b illustrates the influence of factor levels on the water absorption rate. Under factor A, the absorption rate decreases substantially from 25.57% to 12.37%, demonstrating A3’s advantage in minimizing water uptake. Although level B2 exhibits a moderate absorption rate, its substantial density enhancement results in favorable composite performance. For factor C, level C1 shows an absorption rate of 18%, which is comparatively low among its levels and indicates balanced water absorption characteristics. It can be concluded that the factor combination of A3B2C1(B-8) exhibits the most outstanding performance in optimizing the comprehensive properties of the material.

#### 3.1.2. Strength Analysis

**Figure 4:**
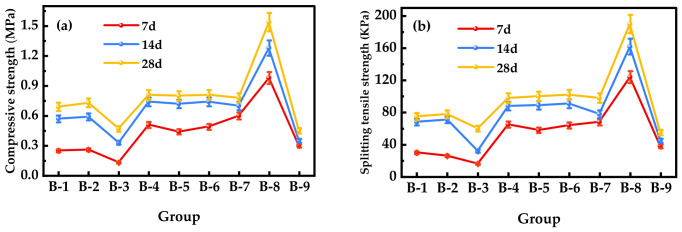
The strength curves at each age of the test groups: (**a**) compressive strength; (**b**) splitting tensile strength.

While compressive strength remains the primary parameter studied for FMLS due to its predominant use as backfill material, this paper investigates its application as a floating landscape carrier. This application necessitates not only compressive strength but also specific performance requirements for splitting tensile strength. Analysis of the strength data at various ages from the orthogonal test groups in Figure 4.

Figure 4a shows that between 7 and 28 days, the compressive strength of group B-3 consistently remains the lowest, while group B-8 consistently exhibits the highest strength. Figure 4b reveals an identical pattern for splitting tensile strength. This is because at early ages, the limited degree of cement hydration yields minimal reaction products, resulting in an incompletely developed cementitious matrix. Consequently, both strength properties remain low with gradual development. By 14 days, advancing hydration produces additional reaction products that partially fill pore spaces and refine the interfacial transition zone between paste and aggregate. At 28 days, near-complete hydration significantly increases structural density, allowing most groups to reach peak strength. Group B-8 achieves the highest strength, likely attributable to its optimized mix proportion or favorable microstructure, which promotes more complete hydration and yields a denser, more homogeneous structure.

**Figure 5:**
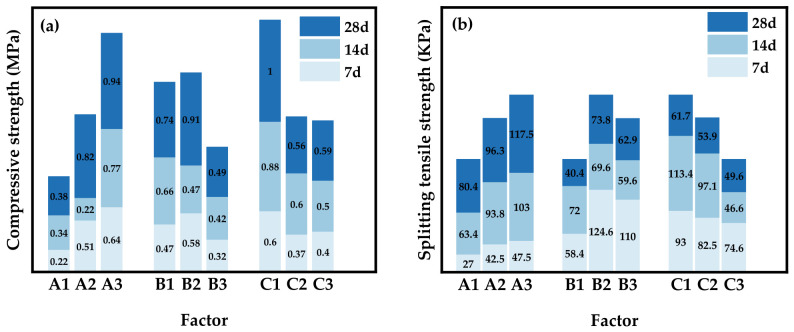
Calculation of extremum difference analysis: (**a**) compressive strength; (**b**) splitting tensile strength.

The extremum difference analysis results are shown in Figure 5. As shown in Figure 5a, level A3 demonstrates a prominent performance in terms of compressive strength at various ages, especially reaching a relatively high level at 28 days, indicating that level A3 has an advantage in enhancing the compressive strength. Level B2 exhibits relatively high and stable average compressive strength values at different ages, particularly showing good performance at 28 days, suggesting that level B2 has an excellent effect on improving the compressive strength. Compared with level C2 and level C3, level C1 has a generally higher average compressive strength at various ages.

In terms of splitting tensile strength, as shown in Figure 5b, level A3 shows a remarkable performance across different ages. It reaches a relatively high value at 28 days, which implies that level A3 has an edge in enhancing the splitting tensile strength. Level B2 also demonstrates relatively high and stable average splitting tensile strength values at various ages. Notably, its performance at 28 days is quite good, signifying that level B2 has an outstanding effect on improving the splitting tensile strength. When comparing level C1, level C2 and level C3, level C1 has a higher average splitting tensile strength at different ages in general. Thus, the factor combination of A3B2C1(B-8) can be considered the optimal combination.

**Figure 6:**
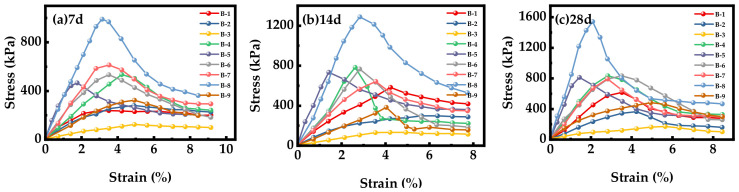
The stress–strain curves at each age: (**a**) 7 d; (**b**) 14 d; (**c**) 28 d.

The stress–strain curves at each age of orthogonal test are shown in Figure 6. From the perspective of comprehensive strength and deformation capacity, the B-8 combination can bear higher stress under the same strain and has better deformation characteristics, so it can be considered that B-8 is the best combination.

Through experiments and analyses in this study, the optimum mixture ratio of the FMLS was determined to be the cement–sand ratio of 0.4, the foam volume content of 10%, and the water–sand ratio of 0.35. Under this mix proportion, the material demonstrates excellent comprehensive performance in terms of density, water absorption rate, and mechanical properties, and meets the requirements of relevant specifications.

### 3.2. Influence Analysis of Adding Various Expansion Agents

Based on group B-8, CaO and MgO expansive agents are added in accordance with the Chinese standard GB/T 23439-2017 [54]. The mix ratio is shown in Table 10.

#### 3.2.1. Fluidity Analysis

**Figure 7:**
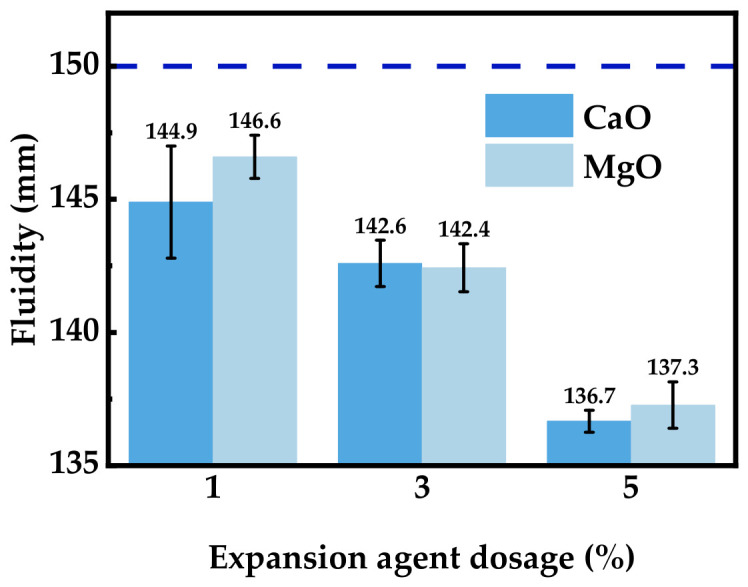
The fluidity changes after adding expansion agents.

Figure 7 shows the changes in the fluidity of the FMLS after adding expansion agents. The dashed line represents the fluidity of the FMLS without the addition of expansion agents. It can be seen from Figure 7 that the effects of the two expansion agents, CaO and MgO, on the fluidity of the FMLS are quite similar at the same dosage. As the dosage of the expansion agent increases, its inhibitory effect on the fluidity of the soil becomes more pronounced. This is because, as the dosage of the expansion agents increases, more hydroxide crystals are generated through the hydration reaction, which reduces the water content in the FMLS to a certain extent, externally manifested as a decrease in fluidity.

#### 3.2.2. Dry–Wet Density and Water Absorption Analysis

**Figure 8:**
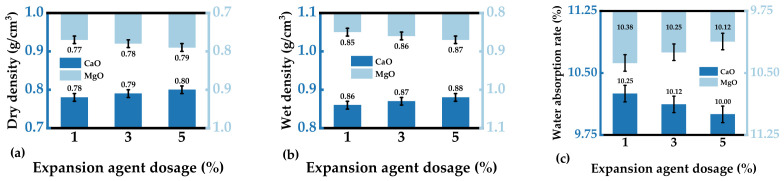
Comparison of analysis results: (**a**) dry density; (**b**) wet density; (**c**) water absorption rate.

It can be seen from Figure 8 that there are changes in the dry–wet density and water absorption of the FMLS after the addition of expansion agents.

The dry–wet density of the FMLS increases after the addition of expansion agents. However, the changes are small. This is because the crystals formed by the hydration reaction of the expansion agent fill the voids between the soil particles and bubbles, which makes the soil structure denser. In the dry state, the content of solid substances per unit volume increases, thus resulting in an increase in the dry density. The addition of the expansion agent increases the mass of the system, which tends to cause an increase in the wet density. However, the hydration reaction consumes a certain amount of water, and the formed crystals have an impact on the soil structure, limiting the increase in wet density to a certain extent. Therefore, the wet density increases, but the range of increase is relatively small. In terms of water absorption rate, with the increase in the dosage of the expansion agent, the water absorption rate shows an obvious downward trend, and the maximum amplitude of decrease reaches 0.4% of that without the addition of the expansion agent. This is because the Ca(OH)_2_ and Mg(OH)_2_ crystals formed by the hydration of the expansion agent fill the pores, reduce the porosity, and hinder the entry of water.

#### 3.2.3. Strength Analysis

**Figure 9:**
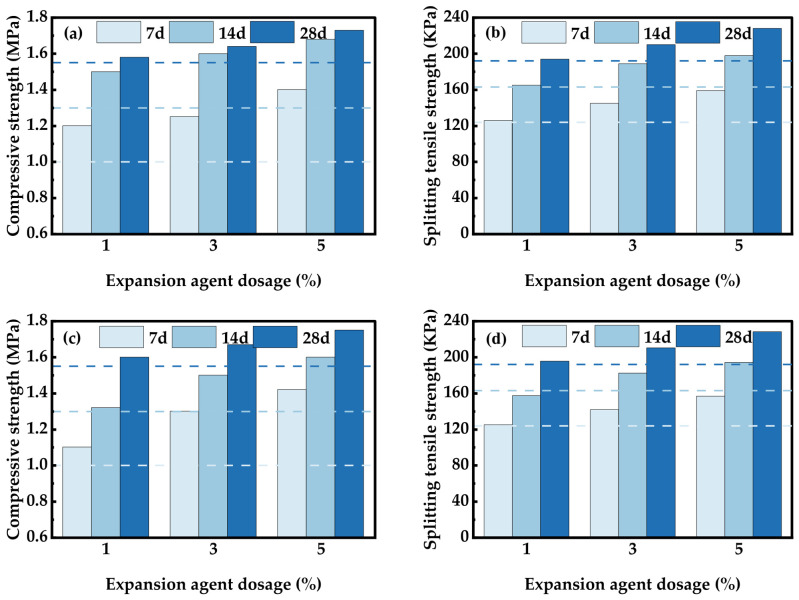
The strength of the optimum mixture ratio with different expansion agent dosages: adding CaO expansion agent (**a**,**b**); adding MgO expansion agent (**c**,**d**).

Figure 9 demonstrates the strength of the FMLS with different dosages of CaO and MgO, where the dashed lines represent the intensity values for group B-8. Comparative analysis reveals nearly identical strength modification effects for both expansive agents. However, distinct hydration mechanisms lead to different temporal strength development patterns. For the CaO expansive agent, compressive strength increases significantly from 7 to 28 days. While its rapid early hydration initially compromises structural density, subsequent hydration products fill pores, enhancing strength. Nevertheless, the intense early hydration can generate localized expansive stresses, partially constraining strength gain. In contrast, the MgO expansive agent hydrates slowly, enabling sustained expansion over a longer period. This results in more uniform shrinkage compensation and microstructural refinement. Consequently, at equivalent dosages, the MgO expansive agent provides marginally superior 28-day compressive strength enhancement than the CaO expansive agent. Regarding splitting tensile strength, the CaO expansive agent exhibits a gradual strength increase from 7 to 28 days. Its early expansive hydration can induce internal microcracks, temporarily limiting initial strength, with subsequent hydration gradually repairing these defects.

The improvement caused by expansive agents can be attributed to both chemical filling and shrinkage-compensation effects. After CaO or MgO reacts with water, Ca(OH)_2_ or Mg(OH)_2_ crystals are formed within the pore system of FMLS. These hydration products partially fill the capillary pores and inter-particle voids, thereby refining the pore structure and increasing the compactness of the matrix. Meanwhile, the micro-expansion generated during hydration can compensate for drying shrinkage and reduce the formation of shrinkage-induced microcracks. Compared with CaO, MgO hydrates more slowly and produces a more gradual expansion effect, which is beneficial for long-term volume stability and internal stress regulation. Therefore, MgO-modified FMLS shows better suitability for ecological floating landscapes, where long-term dimensional stability and resistance to water-induced deterioration are more important than rapid early strength development.

### 3.3. Influence Analysis of Adding Different Kinds of Fibers

The fiber content proportioning was designed as shown in Table 11 to study the effect of fiber parameters on material properties.

#### 3.3.1. Dry–Wet Density and Water Absorption Analysis

**Figure 10:**
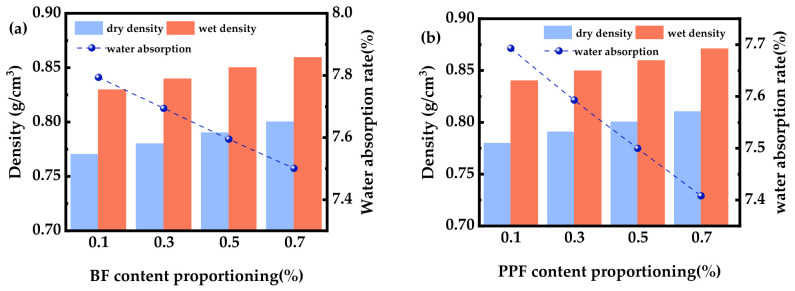
Results of water absorption properties after fiber incorporation: (**a**) BF and (**b**) PPF.

As depicted in Figure 10, upon the incorporation of polypropylene fiber (PPF) and basalt fiber (BF), the dry density exhibits a significantly positive correlation and increases. At a low dosage, the wet density is lower than that in the scenario without fiber addition. This is because the addition of fibers optimizes the uniformity of foam distribution. Consequently, when the specimen is immersed in water, water only penetrates the surface, and the increase in internal water content is limited, leading to a significant reduction in the water absorption rate of the material. In terms of strength performance, by comparing the test results with the Chinese specifications JG/T 266-2011 [55] and CJJ/T 177-2012, it is found that when the content proportioning of BF exceeds 0.5%, and the content proportioning of PPF exceeds 0.3%, the compressive strength fails to meet the requirements specified in the standards.

#### 3.3.2. Strength Analysis

**Figure 11:**
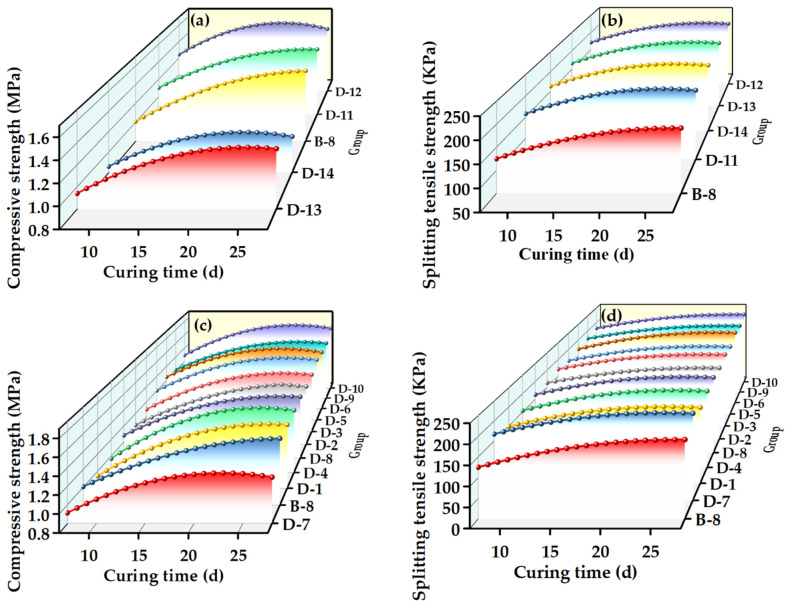
Mechanical properties: PPF-reinforced (**a**,**b**) and BF-reinforced (**c**,**d**).

Figure 11 illustrates the strength test results of FMLS at different curing times after fiber addition. Evidently, fiber incorporation significantly enhances the splitting tensile strength of the FMLS, but the increase in compressive strength is minimal. Notably, among all PPF-reinforced groups, the specimen of group D-12 with 0.3% PPF content exhibits the highest compressive strength, while a significant decline occurs when PPF dosage exceeds 0.3%. For BF-reinforced groups, group D-10 with 0.5% BF content demonstrates the most pronounced increase in compressive strength, whereas the enhancement effect diminishes when the BF dosage exceeds 0.5%. Results of splitting tensile strength tests further confirm the existence of an inflection point in fiber dosage, beyond which the reinforcing effect plateaus or even declines. Specifically, group D-12 with 0.3% PPF achieves the highest splitting tensile strength, representing a 48% increase compared to the fiber-free control group. However, when PPF content exceeds 0.3%, the increment in splitting tensile strength decreases substantially. BF exhibits the same pattern, with inflection point content at 0.5%. Collectively, these findings indicate that the optimal dosages of PPF and BF in foamed mixture lightweight soil are 0.3% and 0.5%, respectively, at which the material strength properties are maximized.

The reinforcing effect of fibers is mainly related to crack-bridging, stress transfer, and pore-structure regulation. Properly dispersed fibers form a three-dimensional network within the cemented dredged-soil matrix, which can restrain the initiation and propagation of microcracks under loading. This mechanism is particularly effective for improving splitting tensile strength because tensile failure is more sensitive to crack growth than compressive failure. However, excessive fiber content may reduce workability, disturb foam stability, and introduce weak interfaces or fiber agglomeration, resulting in reduced compactness and lower strength. Therefore, the reinforcing efficiency of fibers depends on a balance between crack-bridging benefits and the negative effects caused by poor dispersion and increased interfacial defects. In this study, the optimal fiber content reflects this balance.

Integrating the aforementioned findings on compressive and splitting tensile strengths, the optimal dosages of PPF and BF in the FMLS are determined to be 0.3% and 0.5%, respectively. At these optimal concentrations, PPF demonstrates superior enhancement of the splitting tensile strength of the FMLS compared to BF. Specifically, the percentage increase in splitting tensile strength achieved by PPF exceeds that of BF, suggesting that PPF possesses a more pronounced reinforcing effect in improving the tensile behavior of the FMLS. This superiority implies that PPF is a more suitable choice for enhancing the splitting tensile strength properties of the FMLS.

#### 3.3.3. Dry Shrinkage and Toughness Analysis

**Figure 12:**
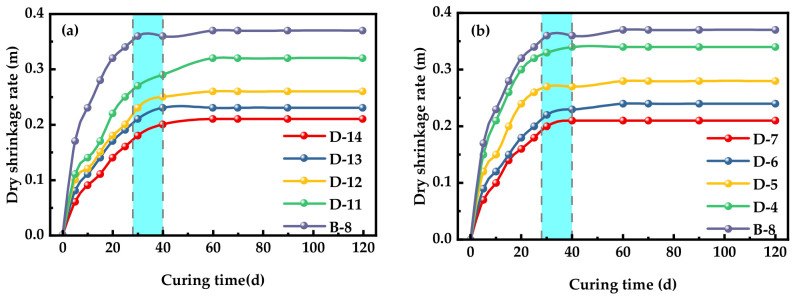
Dry shrinkage-age curve after fiber incorporation: (**a**) PPF-reinforced and (**b**) BF-reinforced.

The dry shrinkage rate variations in fiber-reinforced FMLS are shown in Figure 12. An in-depth analysis showed that the curing age of 28 to 40 days is the key inflection point in the shrinkage curve with the same fiber length. Fibers significantly suppress the shrinkage characteristics of the FMLS. As the PPF content increases from 0.1% to 0.7%, the decrease in the dry shrinkage rate at 120 days of curing exhibits a stepwise increase. At the same fiber dosage, PPF demonstrates a more significant inhibitory effect on the shrinkage rate than BF, underscoring its superior performance in reducing the dry shrinkage of the FMLS.

**Figure 13:**
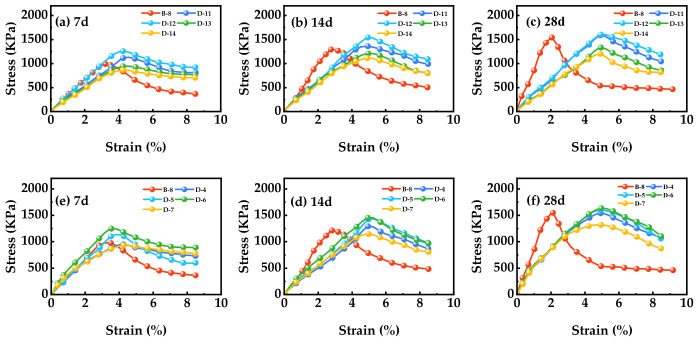
Stress–strain curves of FMLS specimens with different fibers: (**a**–**c**) PPF-reinforced; (**d**–**f**) BF-reinforced.

Figure 13 presents the stress–strain curves of FMLS specimens with different fibers. It can be seen that at the age of 7 days, the peak strain of PPF-reinforced FMLS specimens with different dosages remains stable at approximately 4.2%. This is because the low density of PPF enables it to be uniformly distributed in the matrix of foamed mixed lightweight soil, effectively inhibiting local changes in strain and stabilizing the peak strain. When the age is extended to 14 days, the peak strain of all specimens remains at approximately 4.9%. At this time, the strength of the fiber-reinforced group is significantly improved, indicating that the toughness of the foamed mixed lightweight soil is improved. The result highlights the positive effect of fiber bridging on the ductility of the material. At 28 days, compared with other specimens, the peak stress and ultimate strain of D-12 are more obvious, indicating that the fiber content of D-12 is more ideal and the reinforcement effect is more significant. The specimens added with BF show similar laws to those added with PPF. However, at the curing age of 7 days, the strain at the maximum stress point of D-4 and D-7 groups with BF contents of 0.1% and 0.7% increases to about 4.2%. However, their peak strengths are lower than those of the control group B-8. This is because a small amount or an excessive amount of fibers leads to poor fiber dispersion, resulting in an increase in internal defects of the matrix. When the fiber content is 0.5% (group D-6), the peak strain shifts forward to 3.5%, and the strength increases slightly. When the age is extended to 14 days, the peak strain of all specimens shifts backward. It is worth noting that only the strength of group D-7 is lower than that of the control group, indicating that when the BF content is 0.7%, the strength of the material decreases. At 28 days, the peak strain stabilizes at 4.9%. This result fully verifies the long-term effectiveness of BF in enhancing the toughness of FMLS. Compared with the control group, the strength of group D-6 increases by 6.8%, while the stress peaks of groups D-4 and D-7 decrease significantly. This indicates that the material performance reaches the optimal level when the BF content is 0.5%.

The above analysis shows that the volume dosage of BF should not exceed 0.5% and that of PPF should not exceed 0.3%. However, for the same length and volume dosage, PPF is better for improving the performance of the FMLS.

#### 3.3.4. SEM Analysis

**Figure 14:**
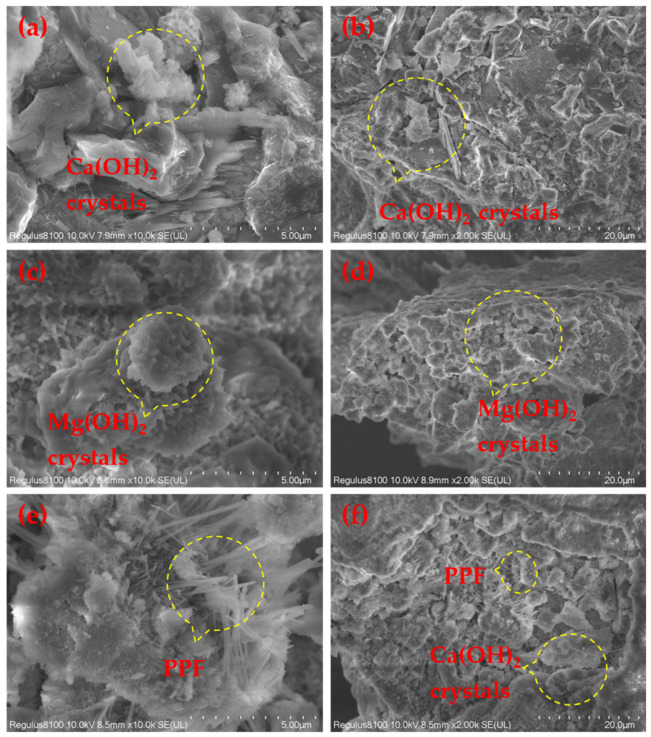
SEM micrographs: group B-8 for (**a**,**b**); group C-6 for (**c**,**d**); group D-12 for (**e**,**f**).

Figure 14 shows the micro-morphological map of group B-8, C-6, D-12, where (a), (b), (c), (d), (e), and (f) are the corresponding micro-morphological maps of 5 μm and 20 μm of the curing age of 28 days, respectively. As can be observed from Figure 14a, the small flocculent structures represent Ca(OH)_2_ crystals formed during cement hydration, while some needle-like and rod-like products correspond to other hydration products. Additionally, a small number of Ca(OH_)2_ crystals are distributed on the sand surface. This phenomenon may be attributed to the fact that during the preparation of the FMLS, small and dense foam clusters coalesce into larger bubbles as a whole, as depicted in Figure 14b. Figure 14c shows the SEM micrograph of the C-6 specimen containing MgO expansive agent after 28 days of curing. Compared with the B-8 group, more plate-like or flocculent products can be observed on the surface of the dredged soil particles. These products may be related to the hydration of MgO and the formation of Mg(OH)_2_-like hydration products. As shown in Figure 14d, the surface coverage of hydration products in the C-6 group appears to be higher than that in the B-8 group, and some pores are partially filled by these products. This suggests that the incorporation of an appropriate amount of MgO expansive agent may promote microstructural densification by filling voids and improving particle bonding.

Figure 14e shows the electron micrograph of the D-12 group specimen with PPF after 28 days of curing. As shown in Figure 14f, upon incorporation of PPF, the fibers interlock to form a specific network architecture, which tightly bridges the surrounding Ca(OH)_2_ crystals and substantially enhances the FMLS internal integrity. This demonstrates that PPF serves as a skeletal reinforcement, effectively reducing the population of macropores and facilitating a more homogeneous pore distribution. Furthermore, the fibers exhibit the capability to impede crack propagation and strengthen interparticle bonding, thereby leading to a more compact microstructure of the FMLS.

SEM analysis reveals that the controlled incorporation of expansion agents and fibers effectively densifies the microstructure and enhances interfacial bonding in FMLS. This microstructural refinement through optimal additive dosage consequently improves the material’s macroscopic performance, demonstrating the effectiveness of this modification approach.

Overall, the SEM observations suggest that the incorporation of MgO expansive agent and PPF can improve the microstructural compactness of FMLS to a certain extent. The MgO expansive agent may contribute to pore filling through the formation of hydration products, while PPF may enhance the fiber–matrix interaction through bridging and crack-arresting effects. These microstructural observations are generally consistent with the improvement in macroscopic mechanical performance. However, because SEM analysis mainly provides qualitative morphological evidence, further studies involving XRD, EDS mapping, mercury intrusion porosimeter, or quantitative image analysis are still needed to more accurately identify hydration products, quantify pore refinement, and clarify the fiber–matrix interaction mechanism.

#### 3.3.5. Correlation Analysis

**Figure 15:**
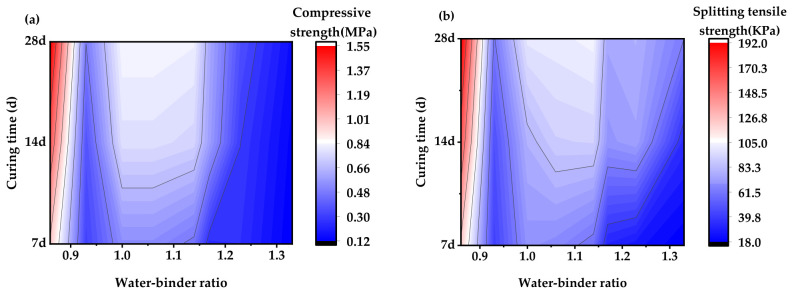
Effect of water–binder ratio on properties of FMLS: (**a**) compressive strength and (**b**) splitting tensile strength.

The analysis of water–binder ratio, curing time, and compressive strength is presented in Figure 15a. At identical curing ages, compressive strength increases with decreasing water–binder ratio. This occurs because lower water–binder ratios increase the relative proportion of cementitious materials, enhancing particle coating and bonding to form a denser microstructure. When the water–binder ratio remains constant, compressive strength progressively develops as curing time extends from 7 to 28 days. This strength gain results from continuous hydration of cementitious materials, where hydration products fill pores and densify the internal structure. Contour lines in Figure 15a exhibit a non-uniform distribution. Regions with closely spaced contours indicate high sensitivity of compressive strength to variations in both water–binder ratio and curing time, demonstrating steep strength gradients. Particularly, minor water–binder ratio changes significantly affect strength in these zones. Conversely, widely spaced contours denote low sensitivity, where strength changes gradually with modified parameters. Analysis of Figure 15b reveals that at constant curing durations, decreasing the water–binder ratio reduces concrete porosity and improves structural homogeneity. This enhanced microstructure better resists tensile stresses, effectively suppressing crack initiation and propagation, thereby increasing splitting tensile strength. When the water–binder ratio remains constant, prolonged curing promotes more complete hydration reactions. This strengthens internal bonding and improves resistance to tensile failure, consequently enhancing splitting tensile strength. Contour distribution analysis shows densely spaced contours in regions with low water–binder ratios and extended curing periods. This indicates high sensitivity of splitting tensile strength to variations in both parameters, where minor changes significantly affect strength. Conversely, sparse contours in areas with high water–binder ratios and short curing durations reflect lower sensitivity, demonstrating gradual strength changes with parameter variations.

### 3.4. Engineering Applicability and Future Research

The modified FMLS developed in this study shows potential for ecological floating landscape applications because it combines low density, acceptable mechanical strength, reduced water absorption, and the reutilization of waste dredged soil. Compared with conventional floating landscape substrates, the proposed material can provide a lightweight cemented matrix with improved dimensional stability and crack resistance. The use of dredged soil also contributes to waste valorization and reduces the demand for natural mineral resources.

Nevertheless, practical engineering applications require further verification under more complex environmental conditions. In actual floating landscape systems, the material may be exposed to long-term immersion, cyclic wetting–drying, temperature variation, biological activity, wave disturbance, and possible chemical attack from polluted water. These factors may affect pore stability, strength retention, shrinkage behavior, and durability. Therefore, future studies should focus on long-term immersion tests, wetting–drying cycle resistance, freeze–thaw durability where applicable, leaching behavior, plant-root interaction, and pilot-scale floating landscape tests. In addition, large-scale production, pumping performance, construction efficiency, cost analysis, and environmental safety assessment should be further evaluated before field application.

## 4. Conclusions

In this study, FMLS suitable for ecological floating landscapes were developed using dredged soil from the lower reaches of the Yangtze River. The optimum mixture ratio was determined based on the physical–mechanical property indexes of compressive strength, dry–wet density, water absorption, and fluidity of FMLS. On this basis, the effects of adding different expanding agents and fibers on the FMLS properties were investigated, and the microstructures of FMLS with different expanding agents and fibers were studied using SEM methods. Finally, a correlation analysis was carried out between water–binder ratio, curing time and strength. The analysis of the test results led to the following conclusions:

(1) The preparation of FMLS from dredged soil suitable for ecological floating landscapes is a new way of efficient resource utilization of dredged soil, which not only reduces the environmental pressure caused by traditional disposal methods but also saves sand resources and has significant economic and environmental benefits.
(2) Through orthogonal experiments, the optimal mixture ratio of FMLS is determined as follows: a cement–sand ratio of 0.4, a foam volume content of 10%, and a water–sand ratio of 0.35. Under this ratio, FMLS exhibits excellent comprehensive performance, including a dry density of 0.77 g/cm^3^, a wet density of 0.84 g/cm^3^, a water absorption rate of 10.4%, and a 28-day compressive strength of 1.55 MPa, fully meeting the requirements of relevant specifications.
(3) Both CaO and MgO expansion agents impact the properties of FMLS. Their addition progressively reduces fluidity with increasing dosage, induces marginal increases in dry–wet density, and significantly decreases water absorption. A 5% MgO dosage achieves a maximum 0.4% reduction in water absorption rate relative to the control group. In terms of strength, MgO expansive agent is more suitable as a strength-modifying material for ecological floating landscapes.
(4) By analyzing the FMLS incorporated with different kinds of fibers, it is found that fibers improve the properties of FMLS. Specifically, PPF outperforms BF in improving splitting tensile strength and reducing dry shrinkage, which can be attributed to PPF’s lower density, facilitating homogeneous distribution.
(5) SEM analysis showed that the right amount of expansion agents and fibers effectively densifies the microstructure and enhances interfacial bonding in FMLS. The correlation analysis shows that the water–binder ratio significantly affects the strength properties of FMLS.

However, the study is still in its infancy, and there are still deficiencies, such as not considering the effect of the combined modification of expansion agents and fibers, and the lack of sufficient microscopic testing. This may have a different effect on the stability of the FMLS and its strength enhancement. Our future research will focus on the effect of the combined modification of FMLS by expansion agents and fibers, and further studies on the effect of the marine environment on FMLS.

## Figures and Tables

**Table 1:** Summary of previous studies and research gaps related to lightweight geomaterials and dredged-soil-based FMLS.

Research Topic	Representative Studies	Main Findings	Research Gaps
Foam lightweight soil using supplementary materials	Zhang et al. [31]; Que et al. [32]	Foam lightweight soil has been developed with fly ash or tailings sand for subgrade and embankment applications. These studies confirmed the feasibility of using solid wastes to regulate density, strength, durability, and microstructure.	Existing studies mainly focus on road embankment or subgrade filling. The coupled requirements of floating landscape carriers, including low density, water stability, tensile resistance, and ecological applicability, remain insufficiently addressed.
Fiber-reinforced lightweight soil	Ren et al. [33]; Jiang et al. [34]; Li et al. [35]	Natural fibers, polypropylene fibers, and other reinforcing fibers can improve crack resistance, toughness, and tensile-related properties of lightweight soils.	The optimal fiber type and dosage for dredged-soil-based FMLS under aquatic service conditions are still unclear.
Dredged sediment reused in cementitious materials	Amar et al. [36]; Zentar et al. [37]	Dredged sediments can be reused as supplementary cementitious materials or stabilized with cementitious binders after proper characterization and treatment.	Most studies focus on cement replacement, road construction, or stabilization/solidification. Few studies convert dredged soil into low-density foamed geomaterials for ecological floating structures.
Dredged sediment-based foamed concrete	Shi et al. [38]; Kou et al. [39]	River or lake dredged sediments have been used to prepare ultra-lightweight or functional foamed concrete, showing potential in sustainable construction materials.	These studies mainly target building insulation, acoustic performance, photocatalytic function, or wall materials, rather than ecological floating landscape carriers.
Expansive-agent-modified cementitious materials	Zhang [40]	MgO expansive agents can compensate for shrinkage, modify hydration behavior, and improve crack resistance in cement-based materials.	The effects of CaO and MgO expansive agents on pore filling, water absorption, and strength development of dredged-soil-based FMLS remain insufficiently studied.
Substrate materials for ecological or constructed wetland systems	Mlih et al. [41]; Wei et al. [42]	Lightweight porous substrates such as LECA and other modified materials can support pollutant removal, hydraulic conductivity, plant rooting, and biofilm growth in wetland systems.	Existing substrate studies emphasize water purification, but rarely combine ecological function with structural performance and dredged soil resource utilization.
Overall research gap		Lightweight geomaterials, dredged sediment reutilization, fiber reinforcement, and expansive-agent modification have each been investigated separately.	A systematic material design that integrates dredged soil reutilization, FMLS lightweight structure, fiber reinforcement, expansive-agent pore regulation, and ecological floating landscape application is still lacking.

**Table 2:** Dredging soil major components.

Components	SiO_2_	Al_2_O_3_	Fe_2_O_3_	CaO	MgO	K_2_O	Na_2_O	TiO_2_	SO_3_
Content: %	63.73	14.33	4.82	8.54	3.42	2.70	1.36	0.658	0.04

**Table 3:** Different foaming agent performance parameters.

Foaming Agent	Subsidence Distance: mm/L	Water Secretion: g/h	Dilution Ratio	Foaming Ratio
Animal protein	7	80	20	27
Plant protein	5	20	60	20
Composite	6	40	30	35

**Table 4:** Basic properties and composition of the expansion agent.

Expansion Agent	Content: %	Burn Weightlessness: %	Ethanol Insoluble Substance: %	Fineness: Mesh
CaO	98.6	2	0.05	800
MgO	98	2	Hydrochloric acid insoluble substance: %	water-soluble material
			0.01	0.5

**Table 5:** Performance parameters.

Material	Length: mm	Tensile Strength: MPa	Density: g/cm^3^	Elastic Modulus: GPa	Fiber Diameter: μm
Polypropylene fiber	9	486	0.91	4.8	18–48
Basalt fiber	6–12	1050	2.65	7.6	17

**Table 6:** Pre-test design.

Group	Water–Sand Ratio	Cement–Sand Ratio
A-1	0.2	0.2
A-2	0.25	0.3
A-3	0.3	0.4
A-4	0.35	0.4
A-5	0.4	0.4

**Table 7:** Levels of orthogonal experimental design factors.

Level	Factor		
	Cement–Sand Ratio (A)	Foam Volume Content (B)	Water–Sand Ratio (C)
1	0.3	8%	0.35
2	0.35	10%	0.37
3	0.4	12%	0.4

**Table 8:** Orthogonal experimental design mix ratios.

Group	Cement–Sand Ratio	Foam Volume Content	Water–Sand Ratio
B-1	A1	B1	C1
B-2	A1	B2	C2
B-3	A1	B3	C3
B-4	A2	B1	C2
B-5	A2	B2	C3
B-6	A2	B3	C1
B-7	A3	B1	C3
B-8	A3	B2	C1
B-9	A3	B3	C2

**Table 9:** Wet-dry density and water absorption of orthogonal test.

Group	Dry Density: g/cm^3^	Wet Density: g/cm^3^	Water Absorption Rate: %
B-1	0.56	0.67	19.6
B-2	0.54	0.71	31.5
B-3	0.43	0.54	25.6
B-4	0.59	0.65	10.2
B-5	0.76	0.85	11.8
B-6	0.50	0.62	24.0
B-7	0.67	0.72	7.5
B-8	0.77	0.84	10.4
B-9	0.52	0.62	19.2

**Table 10:** Mix ratio after adding expansion agents.

Group	Water–Sand Ratio	Cement–Sand Ratio	Foam Volume Content: %	CaO Volume Content: %	MgO Volume Content: %
C-1	0.35	0.4	10	1%	/
C-2				3%	/
C-3				5%	/
C-4				/	1%
C-5				/	3%
C-6				/	5%

**Table 11:** Design of fiber content proportioning.

Group	Water–Sand Ratio	Cement–Sand Ratio	Foam Volume Content: %	Fiber Type	Length: mm	Fiber Volume Content: %
D-1	0.35	0.4	10	BF	6	0.10
D-2				BF	6	0.30
D-3				BF	6	0.50
D-4				BF	9	0.10
D-5				BF	9	0.30
D-6				BF	9	0.50
D-7				BF	9	0.70
D-8				BF	12	0.10
D-9				BF	12	0.30
D-10				BF	12	0.50
D-11				PPF	9	0.10
D-12				PPF	9	0.30
D-13				PPF	9	0.50
D-14				PPF	9	0.70

## Data Availability

The original contributions presented in this study are included in the article. Further inquiries can be directed to the corresponding author.
